# The Effects of Music-Based Patterned Sensory Enhancement on Motor Function: A Scoping Review

**DOI:** 10.3390/brainsci15070664

**Published:** 2025-06-20

**Authors:** Chantelle C. Caputo, Marija Pranjić, Yuko Koshimori, Michael H. Thaut

**Affiliations:** 1 Music and Health Science Research Collaboratory, University of Toronto, Toronto, ON M5S 1C5, Canada; chantelle.whiteside@mail.utoronto.ca (C.C.C.); michael.thaut@utoronto.ca (M.H.T.); 2 Division of Developmental Medicine, Boston Children’s Hospital, Harvard Medical School, Boston, MA 02115, USA; 3 Faculty of Medicine, Institute of Medical Science & Rehabilitation Sciences Institute, University of Toronto, Toronto, ON M5S 1C5, Canada

**Keywords:** neurologic music therapy, patterned sensory enhancement, rhythmic auditory stimulation, rhythmic auditory cueing, music-based exercise, motor function

## Abstract

**Background/Objectives:** Patterned Sensory Enhancement (PSE), a Neurologic Music Therapy technique, utilizes rhythm and other musical elements to facilitate functional movement in diverse clinical populations. This scoping review is the first to systematically synthesize the current evidence surrounding PSE’s use and its effects on motor function across various populations in order to evaluate its therapeutic potential, identify gaps in the existing literature, and guide future research efforts. **Methods**: A literature search was conducted across five major databases (MEDLINE, Embase, PsycINFO, CINAHL, and Scopus) in accordance with the PRISMA-ScR guidelines. **Results**: From 1018 screened articles, 15 met the inclusion criteria. PSE has been demonstrated as effective across clinical populations, including Cerebral Palsy, stroke, Parkinson’s Disease, and psychiatric conditions. However, the results for studies on geriatric populations remain inconsistent. Despite the variability in the outcome measures and movement types assessed, PSE is consistently supported as an effective approach for enhancing motor function. However, to date, only a small number of studies across populations have been conducted. **Conclusions**: This scoping review suggests that PSE holds significant potential for improving motor function across a range of clinical populations. Further research is needed to explore the long-term effects, use standardized terminology, and identify the optimal implementation strategies tailored to the unique needs of different populations to maximize its therapeutic benefit.

## 1. Introduction

Research into the neuroscience of music has highlighted the potential of music-based interventions to influence non-musical functions by enhancing brain recovery processes [1,2,3,4,5]. Notably, the integration of applied music neuroscience with movement has proven effective in therapeutic and rehabilitative settings, particularly in improving motor outcomes [6,7].

Neurologic Music Therapy (NMT), a clinical application of music neuroscience research, utilizes standardized sensorimotor interventions to support the rehabilitation and recovery of motor function using music, including rhythm and other pitched musical elements [8], and has been demonstrated as an effective and feasible form of treatment across functional domains and clinical diagnoses [9,10]. The effectiveness of NMT for improvement in motor function is primarily a result of the close functional connectivity between auditory and motor areas throughout the central nervous system, as demonstrated in neurophysiological and neuroimaging studies [11,12]. Precise and immediate coupling between auditory and motor-associated brain regions, including the basal ganglia and cerebellum, has been demonstrated with rhythmic auditory cueing.

Behaviorally, isochronous rhythmic auditory cues, such as metronome clicks or rhythmically salient music, provide regular and predictable temporal information and foster auditory–motor entrainment in a feedforward interaction, enhancing the speed, stability, and efficiency of motor actions [2,5]. For the upper extremities (UEs), rhythmic cues drive movement, impacting the onset, duration, and variability of electromyography patterns [13]. Additionally, the use of external rhythmic auditory cues has been shown to improve hand and arm movements in individuals with Parkinson’s disease (PD) [14,15,16,17]. For the lower extremities, the application of Rhythmic Auditory Stimulation (RAS) has shown great promise in improving gait, postural stability, and other motor functions [18,19,20,21,22] by utilizing external rhythmic cues to enhance motor coordination and timing, stabilizing the spatiotemporal patterns through auditory–motor coupling among neurological populations such as PD and stroke [23,24,25,26,27].

### 1.1. Patterned Sensory Enhancement

Unlike RAS that addresses purely gait and lower extremity function, Patterned Sensory Enhancement (PSE), another NMT clinical intervention, addresses whole-body neuromotor coordination, both of which can be facilitated with the purely rhythmic cues of a metronome, or layered with other musical elements. PSE uses multiple musical elements including rhythm, melody, harmony, dynamics, and acoustics to provide temporal, spatial, and force cues to facilitate volitional, functional movements that are not necessarily intrinsically rhythmic [14]. These kinematic patterns and sequences are translated into sonified stimuli for sound, auditory guidance, and feedback to facilitate the desired movement [14]. For instance, a Neurologic Music Therapist may use tempo, meter, and rhythmic patterns can help to control the speed and timing of a movement, while the variations in pitch or direction of a melody can guide the direction and range of a motion. Additionally, the use of specific dynamics can prime increasing muscle force through volume changes, while a harmony from dissonance to consonance can facilitate tension and release. These musical components work together to create a predictive sound pattern that guides the execution of functional movements [14]. Because PSE is function-based and not population-specific, it is appropriate to target physical goals across a variety of neurologic populations ranging from children to geriatric patients, with the unique patient and population impairments guiding the clinician in intervention design [14]. Clinical guidelines for therapy as suggested by the “NIH Music-Based Intervention Toolkit” may generally apply to PSE, outlining the important consideration of determining which music intervention is most appropriate for the client, ensuring that their needs will be addressed [27]. Emerging clinical trials are investigating the efficacy of PSE across various clinical populations and settings, underscoring its broad therapeutic potential.

PSE is grounded on sonification principles, which use sound to convey non-sound information [28,29]. As it relates to motor function, the desired movement pattern is mapped into sound parameters (typically pitch, volume, or rhythm) and delivered through musical elements (like melody, chords, tones, and rhythm) to target specific motor goals through a feedback interaction [29]. In addition to this feedback interaction, PSE can also elicit feedforward interactions by using sensorimotor integration of auditory–motor control to drive the desired motor output, particularly by providing precise priming and timing information [14]. Sonification maps physiological and physical data onto psychoacoustic parameters to facilitate on- and/or off-line access to biomechanical information [28,30,31,32,33,34,35]. While these mechanisms that also underly RAS continue to apply to PSE, the primary difference is that RAS is applied to biologically rhythmic movement (gait) while PSE is used for movements that are not inherently rhythmic in nature [14].

While rhythm is an important component of both PSE and RAS, the use of purely rhythmic cues for UEs is still considered PSE, as long as it addresses full-body neuromotor coordination, despite an inconsistency in the literature terminology [8,36].

### 1.2. Aims and Research Question

The aim of this scoping review was to synthesize the existing research on PSE, categorized by the diverse populations in which PSE has been studied. The research question addressed was as follows: “what is the scope of the existing research on PSE, particularly in terms of its efficacy and application across diverse clinical populations?” By addressing this, there lies potential to further lay the groundwork for future research and clinical applications.

## 2. Materials and Methods

The scoping review was performed in compliance with the Joanna Briggs Institute methodological framework [37] using the Preferred Reporting Items for Systematic Reviews and Meta-Analyses Extension for Scoping Reviews (PRISMA-ScR) criteria: Checklist and Explanation guidelines [38,39]. The protocol was registered with the Open Science Framework on 2 August 2023 and is available online at https://doi.org/10.17605/OSF.IO/YMXEK. We aimed to address the following question: what is known in the literature about the efficacy of PSE on motor function across clinical populations?

### 2.1. Eligibility Criteria

Articles were eligible if they (1) involved the use of PSE to enhance motor function in individuals across the lifespan, (2) reported the pre–post PSE outcome measures, (3) were published in English, and (4) were categorized as original research. Given the wide variation in PSE terminology across the literature and a common misuse of the term RAS for UEs, we also included articles that reported on the effects of auditory cueing (rhythmic, melodic, and/or sonified) for the upper extremities. Articles were excluded if they were review papers, book chapters, conference proceedings, or master’s theses, or involved machine use for learning or training. The publication year was not specified as an inclusion/exclusion criterion, in order to capture all the existing research.

### 2.2. Search Strategy

The search was carried out across five databases including MEDLINE (Ovid), Embase (Ovid), PsycINFO, CINAHL, and Scopus for articles published before August 2024. The search strategy was developed using the population, concept, and context (PCC) mnemonic. To define the concept, we used terms such as “Patterned Sensory Enhancement,” “Music-Based Exercise,” “Rhythmic Auditory Cue,” and “Neurologic Music Therapy.” Subject headings were adapted for each database. The population and context were intentionally not specified to capture the full range of publications regardless of the setting (e.g., clinic or research laboratory) and clinical population (e.g., children or adults). The search strategy was developed and performed by two authors (C.C. and M.P.) (see Supplementary Table S1 for the full electronic search strategy).

### 2.3. Screening and Data Extraction

All titles, abstracts, and full-text publications were screened by two independent reviewers (C.C. and M.P.) and any discrepancies in the screening decisions were resolved via consensus. The filtering processes of the search results were completed using the Covidence software. We systematically extracted the following information from the included papers: publication details, sample characteristics (i.e., age, sex, sample size), study design, performance tasks, rhythmic and musical stimuli, outcomes measures, and key findings. Data from the selected studies were collated, summarized, and reported based on the clinical population, study characteristics (including design, performance tasks, outcome measures, rhythmic and musical stimuli, and key findings), efficacy of PSE on motor function, and findings across populations to better understand the impact of PSE.

## 3. Results

**Figure 1:**
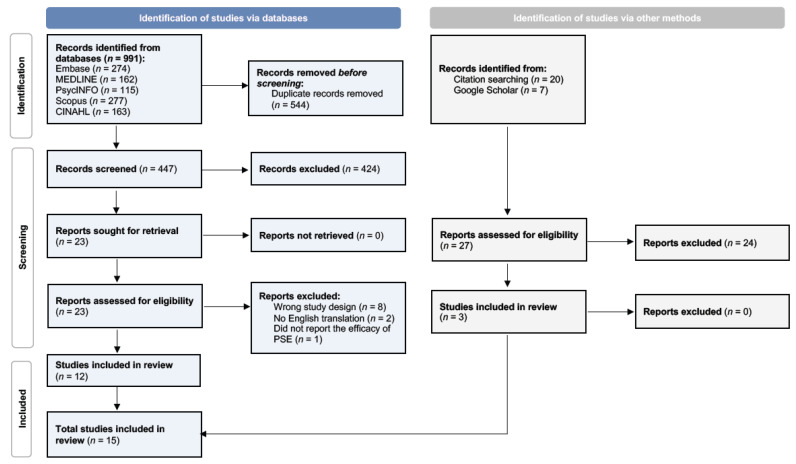
Selection of sources of evidence (PRISMA-ScR Flowchart).

A total of 1018 sources were identified from searches of five electronic databases, citation searching, and Google Scholar (see Figure 1). In Covidence, 447 articles were screened based on the title and the abstract, and 424 articles were excluded due to failing to meet the inclusion criteria. A total of 23 sources remained for full-text review. Of these, 2 studies were not published in English, 1 did not report the efficacy of PSE, and 8 records were review papers or non-peer-reviewed publications. We screened an additional 27 sources identified from citation searching and Google Scholar. Together, 15 studies were considered eligible for this review and are summarized in Table 1. These studies tested the efficacy of PSE in individuals with Cerebral Palsy (*n* = 2), stroke (*n* = 6), Parkinson’s Disease (*n* = 3), geriatric patients (*n* = 2), and psychiatric populations (*n* = 2).

### 3.1. Study Designs

The study designs included randomized controlled trial (*n* = 4), clinical control trial (*n* = 4), and pre-, post-test (*n* = 7). The sample size ranged from 5 to 60 participants, including CP, stroke, PD, geriatrics, and psychiatric conditions. A number of outcome measures were utilized across the studies. Standardized functional assessments were used in 8/15 studies, including the Fugl-Meyer Upper Extremity (*n* = 2), Wolf Motor Function Test (*n* = 2), Action Reach Arm Test (*n* = 1), Purdue Pegboard Test (*n* = 2), Rhomberg’s Test (*n* = 1), Barthel Index (*n* = 1), Gross Motor Function Measure (*n* = 1), and Pediatric Evaluation of Disability Inventory (*n* = 1). Other assessment measures included motion capture kinematics (*n* = 7), a dynamometer for hand grip and muscle strength (*n* = 2), and electromyography for muscle activity of the biceps and triceps (*n* = 2). Considerable heterogeneity of the interventions was noticed across the studies. The interventions were delivered individually (*n* = 10) or to a group (*n* = 5). The musical stimuli varied between live and pre-recorded musical elements, including rhythm solely presented by using a metronome, melody, tune, and music. The duration ranged from brief task repetition to full 45 min sessions, with frequencies of 1 to 21 sessions, spanning 1 day to 6 weeks.

### 3.2. Efficacy of PSE

The efficacy of PSE was assessed across the literature. The following section provides a synthesis of the included studies organized by clinical population, including Cerebral Palsy, geriatrics, Parkinson’s Disease, stroke, and psychiatric conditions. As PSE is function-based and not population-specific, the details of various UE functions are highlighted, including sit-to-stands, activities of daily living, reaching, movements of the shoulder, arm, and hand, and functional UE tasks. The details of the study design, performance tasks, outcome measures, rhythmic and musical stimuli, and key findings are also addressed.

#### 3.2.1. Cerebral Palsy and Spastic Diplegia

Empirical research on the use of PSE in pediatrics has focused on children with Cerebral Palsy (CP) and Spastic Diplegia. Two notable studies have investigated its effects during loaded sit-to-stand (LSTS) exercises, offering valuable insights into PSE’s potential to enhance movement. Using a crossover, within-subject design, Peng et al. [41] studied gender-matched participants (*N* = 23) who were exposed to LSTS conditions: an experimental condition with PSE music, and a control condition without music. The experimental condition used individualized PSE music composed by a music therapist on a Garage Band electric keyboard with a meter of either 2/4, 3/4, 4/4, or 6/4. This involved a rhythmic beat preparation period tailored to the individual baseline movement speed, a harmonic chord progression that cued seat-off, and ascending and descending melodic lines to cue the movement direction. The PSE music was played during the first five repetitions of exercises, followed by three additional repetitions without music. In the no-music condition, all eight repetitions were performed without music. The participants completed the tasks in both conditions in randomized order. The key outcome measures included movement trajectories, velocities, and accelerations, captured using motion capture analysis (Vicon), as well as the grand reaction forces of each foot, recorded via footplates, assessed during both the experimental and control conditions for immediate and continuous effects. The results demonstrated that individualized PSE music led to significantly and immediately improved peak knee extensor power and total extensor power, improved movement smoothness, and reduced movement time, with the positive effects remaining for three following sit-to-stand cycles without music.

The researchers, Wang et al. [40], then further developed this by conducting a randomized controlled trial involving age-matched participants (*N* = 36) equally split into an experimental and control group. Each completed a 6-week, home-based PSE training program, with the experimental group training with prerecorded PSE music, while the control group completed the same exercises without music. The PSE intervention used various musical elements including tempo and meter tailored for individual needs. For example, increasing the length of sustained chords was used to prolong balance, while slower descending scales targeted the improved control of sitting. Primary outcome measures assessed the gross motor capacity and functional strength during standing, walking, running, and jumping, using the Gross Motor Function Measure Dimensions D (13 items) and E (24 items). A Goal Dimension score was derived from the average scores of Dimensions D and E scores, measured at the baseline, after 6-weeks of training, at 6-weeks post-training, and at 12-weeks post-training. The results showed that the PSE group had greater improvements than the no-music group at pre- and post-training and follow-up after 6 weeks. Both groups had increased secondary outcome measures of PEDI mobility, which demonstrated a significant main effect of time, but no significant interaction. There was no significant improvement in the Caregiver Assistance Scale of PEDI mobility in either group. PEDI self-care improved in both groups from the baseline to 6 weeks post-treatment and the baseline to 12 weeks post-treatment but not the baseline to after 6 weeks of treatment. This showed no significant main effects of time, but a significant interaction effect from the baseline to 12 weeks post-treatment with greater improvements in the no-music group. Those who exercised with PSE had statistically significant improvements in their gross motor capacity; however, PSE did not achieve statistically significant improvements in daily functioning, strength, and walking speed. These studies demonstrate a benefit for certain aspects of motor function, such as movement smoothness, speed, and power, and gross motor capacity.

#### 3.2.2. Stroke

Six studies explored the use of PSE for improving upper-extremity motor function in stroke populations. In the first, Kang et al. [49] investigated the effects on shoulder kinematics in individuals with hemiparesis resulting from a first-time ischemic or hemorrhagic stroke (*N* = 18). Utilizing a within-subjects, repeated measures design, this study employed three different cueing conditions: no cue, rhythmic PSE (rhythmic auditory cueing), and melodic PSE (rhythmic melodic auditory cueing). During rhythmic PSE, the participants heard metronomic tones at a frequency of 440 Hz, while melodic PSE involved tones at varying frequencies but maintaining the isochronous timing pattern. The shoulder movements included abduction, holding, and adduction. The participants underwent a movement practice period for the different cues, followed by the experimental phase of three shoulder movement blocks during each cueing condition, with five trials per condition in a randomized order. The kinematic parameters assessed during each of the cueing conditions included range of motion, minimum and maximum Euler angles, movement duration, and root mean square error. The researchers found that during the stationary phase, melodic PSE demonstrated a higher minimum Euler angle and decreased the range of motion when compared to the other cues. Additionally, melodic PSE resulted in a shorter movement time particularly in the stationary phase, as well as a smaller root mean square error in the angle measurements. Both PSE conditions led to enhanced movements across all the kinematic parameters when compared to no cue.

Malcolm et al. [51] employed a pilot, pre-test/post-test design with chronic stroke participants (*N* = 5). The participants were tasked with PSE-RAS reaching between assigned targets using their affected hand. This began at a self-paced baseline speed, and then was synchronized to steady rhythmic beats with targets increasing in distance and changing directions to encourage varied shoulder movements. Onsite or home-based training consisted of 3 h a day, five days a week, for two weeks. Motor control was assessed during the reaching task using kinematic motion analysis, while motor function, capacity, and quality were assessed via the Wolf Motor Function Test (WMFT) immediately before and after the intervention period. The kinematic analysis showed that PSE-RAS during reach tasks led to a significant decrease in trunk compensation, an increase in shoulder flexion, and a slight increase in elbow extension, as well as a significant improvement in movement time and velocity. Additionally, the participants demonstrated significant functional gains on the WMFT.

Kim et al. [50] examined the motion and muscle activation of the elbow during reaching tasks (*N* = 16). This within-subjects design implemented two comparison tasks: with and without PSE-RAS during repetitive forward target reaching with the affected arm at 1 min intervals. Without PSE-RAS, the participants touched the target at a comfortable, baseline pace, and with PSE-RAS involved the same movement in sync with a metronome beat that matched the baseline speed. The condition order was randomly assigned to control for possible order effects. During the tasks, motion analysis measured the movement speed, range, and smoothness, while electromyography (EMG) was used to measure the muscle activity and co-contraction ratios of the affected arm triceps and biceps. Motion analysis demonstrated significantly decreased movement time and number of movement units, and significantly improved elbow extension, while the EMG results demonstrated significantly increased muscle activation in the triceps, and a significantly decreased co-contraction ratio.

Tian et al. [48] conducted a randomized controlled pilot study with participants (*N* = 30) matched for age and time post-stroke. Over 4 weeks, everyone maintained 30 min each of physical and occupational therapies daily. The experimental group (*n* = 15) received an additional 30 min daily of training with rhythmic cues, while the control group (*n* = 15) received an additional 15 min daily each of physical and occupational therapies. The movements during PSE-RAS included shoulder, elbow, forearm, wrist, and reach exercises, and functional tasks of holding and moving objects of varying sizes. Assessments were conducted before and after all treatments, including the Fugl-Meyer Upper Extremities (FM-UE), WMFT, and Barthel Index (BI), as well as surface EMG recordings on the hemiparetic biceps and triceps during elbow flexion and extension which measured the co-activation interval and co-contraction index. Significant improvements were found within and between the groups after the treatments, with the experimental group demonstrating higher WMFT and BI scores. Statistically significant improvements were found in the co-activation interval between the groups after training, but not in co-contraction.

Kalidasan et al. [52] explored the effect of PSE-RAS compared to mirror therapy (MT) and conventional therapy (CT). The participants (*N* = 60) were divided into three equal groups to receive their designated treatment for 20 min per day, five times per week, for 4 weeks. The CT group completed muscle tone normalization exercises, free arm movements, and sensory reeducation to encourage volitional movements; the PSE-RAS group moved in synchrony with a metronome beat during movements like coin stacking, and grasping, releasing, rolling, and squeezing a ball; and the MT group completed arm and hand movements of the affected and unaffected arm simultaneously in front of a mirror. The assessment measures included a handheld dynamometer for hand grip and the Action Reach Arm Test (ARAT) for hand function. They found significant differences between the groups in both their post-test ARAT scores and hand grip, when compared to no significant differences at pre-test. Significant improvements in hand function were demonstrated from pre- to post- in the CT (13.91%), PSE-RAS (46.57%), and MT (25.47%) groups, and in hand grip among the CT (13.55%), PSE-RAS (55.46%), and MT (22.92%) groups.

Chouhan et al. [47] studied the efficacy of both PSE-RAS with CT and visual cueing (VC) with CT on gross and fine motor activities, as well as comparing the effects of each. The participants (*N* = 45) were randomly and evenly assigned to one of three groups: PSE-RAS with CT, visual cueing with CT, or CT alone. The PSE-RAS group was tasked with moving the affected hand’s fingers between at least two targets and self-paced maximal speed, then in sync with a metronome beat. The VC group picked different colored objects of different shapes and sizes, picked up and transferred a ball, and picked up a pen. Both groups also completed traditional stretching of tightened muscles. The CT group completed stretching of tightened muscles. The assessment method used was the FM-UE. The results demonstrated continuous improvements in FM-UE scores from day 14, 21, and 28 among all three groups. After 1 month of treatment, significant improvements were demonstrated in all three groups. Only PSE-RAS led particularly to improved gross and fine motor FM-UE scores from day 14 to 28. These six studies demonstrate that the use of PSE for upper-extremity movements is an efficacious intervention among stroke populations.

#### 3.2.3. Parkinson’s Disease

Three studies investigated the use of PSE for motor function in PD. In the first, Bukowska et al. [46] investigated the efficacy of music and rhythm on mobility and balance, specifically examining the combined effects of NMT sensorimotor techniques including PSE in a pilot, between-subjects study with age-matched participants (*N* = 55). The experimental group (*n* = 30) participated in 45 min NMT sensorimotor sessions four times a week for 4 weeks. These sessions integrated three motor techniques, PSE, Therapeutic Instrumental Music Playing (TIMP), and RAS, using primarily African and Indian rhythmic music to facilitate the organization and fluency of movement to target activities of daily living (ADLs), balance, pre-gait, and gait training. In contrast, the control group (*n* = 25) was asked to maintain their typical ADLs including position changes, walking, and climbing stairs. Gait parameters were measured using the Optoelectrical 3D Movement Analysis System BTS Smart, while stability was assessed through Computerized Dynamic Posturography CQ Stab using Romberg’s balance test. The Rhomberg’s test demonstrated no significant differences between groups in regard to balance and stability with eyes open and closed. However, the experimental group showed improved stability with eyes open compared to the control group, and improvements in all five test parameters with eyes closed. They determined that PSE and NMT motor techniques may not necessarily influence static stability in PD, but possibly improve eyes closed proprioception.

The second, a study by Fan et al. [45], examined the effect of PSE-RAS on upper-extremity movements during the Purdue Pegboard Test. Age-matched participants (*N* = 46) were tasked with picking up one pin or pin pairs and inserting them in the board with the right hand, left hand, and both hands, first self-paced to determine the 100% baseline movement speed, and then with a metronome beat at increasing tempi of 100%, 110%, and 120%. The results showed that faster PSE-RAS induced faster upper-limb movements, as the 120% speed led to higher pegboard test scores than the other speeds, and the 110% speed led to higher scores than 100%. Task effects were also found, as the right-hand task had higher scores than the left, and the left-hand task had higher scores than the both-handed task.

In the third and most recent study, Smith et al. [44] examined the effects of auditory cueing on upper-extremity movement smoothness and path variance in a within-subject design. PD participants (*n* = 7) and a convenience sample of neurotypical college students (*n* = 10) were exposed to three conditions: no cueing, rhythmic PSE cueing, and sonified PSE cueing. The rhythmic PSE cue involved a metronome set at 70 beats per minute, while sonified PSE used pitched musical and rhythmic cues to facilitate timing, spatial, and force movement requirements during a pre-recorded folk tune at 70 beats per minute with an embedded metronome. The participants performed three 60 s trials of repetitive arm reaching for each cueing condition. To control for order effects, each began with the no cue condition, followed by the remaining conditions in a randomized order. Breaks were provided between trials of the same condition for 30 s and different conditions for 90 s. Kinematic parameters were measured using motion capture analysis to assess the normalized jerk and spatiotemporal index. The PD group showed no significant main effect of cueing condition; however, they did demonstrate a lower normalized jerk with PSE and higher normalized jerk with the auditory cue. Repeated measures ANOVA showed a lower spatiotemporal index than the college group without statistical significance. Among the Parkinson’s group, there was no significant effect of cueing condition, but higher spatiotemporal index mean values in the rhythmic cueing condition and lower spatiotemporal index mean values in the PSE and no cueing conditions. PSE then may improve the movement smoothness among people with PD. Research into the use of PSE for PD has found benefits, particularly for movement smoothness.

#### 3.2.4. Geriatrics

Two studies explored the effects of PSE, focusing on muscle strength and exercise performance in older adults. In the first, Toma et al. [43] studied participants (*N* = 6) with degenerative neuromotor deficits using a within-subjects, pre–post design. The PSE intervention was used as part of a larger music therapy protocol, where the participants engaged in 60 min sessions twice a week for four weeks. Each session began and ended with 15 min of PSE, though the details of the middle 30 min of the music therapy session were not specified. The PSE tasks included hand grip, cued by harmonic chords with tension and resolution, and arms up and down, cued by ascending and descending melodic patterns. Muscle strength was measured using a Takei dynamometer. A secondary outcome measure involved a post-experiment questionnaire administered 6 months later. The researchers found that PSE led to improvements in all the participants. Of the subjects, 71% underwent improved muscle strength greater than 30% from pre- to post-treatment. The post-experiment questionnaire at 6-month follow-up indicated self-reports of increased musculoskeletal relaxation and supported motor task completion.

In the second, O’Konski et al. [42] compared pre-recorded PSE to background big band music during exercise with long-term care residents (*N* = 45) using a within-subjects design. Sessions focused on 19 restorative exercises, taking place twice a week over four weeks, with the final week designated for make-ups. Each participant completed three PSE and three big band music sessions, with one of each condition per week in a counterbalanced order. PSE involved a composition recorded on CD by a Board Certified Music Therapist and Neurologic Music Therapist, while the background music used a CD of big band music, both of which were used consistently across all the sessions. The outcome measures included the number of repetitions, adherence to the modeled movements (synchrony with facilitator), range of motion, and form. PSE led to synchrony in 3/19 exercises, while no other significant differences were found between the two conditions. These studies demonstrate that PSE used among geriatric populations may have some benefit.

#### 3.2.5. Psychiatric Conditions

Two studies have investigated the effects of upper-limb training with PSE-RAS for psychiatric populations, demonstrating an impact on movement speed. The first study, conducted by Wang et al. [54], examined the effect of PSE-RAS on the upper-extremity movement speed in individuals with Schizophrenia Spectrum Disorders (SSDs) (*n* = 30) and gender-matched healthy controls (*n* = 30) in a 2 × 2 mixed design. The participants completed the Purdue Pegboard Test, both as a right-hand task and a simultaneous two-handed task. The participants first completed the task self-paced to establish their baseline performance and speed; then, they were randomly assigned to one of two PSE-RAS speed condition orders: normal followed by fast or fast followed by normal. For each condition, they inserted pins into holes with each metronome beat. Matching the baseline speed, metronome cues were used at 100% for normal and 120% for fast. Those with schizophrenia-related disorders had lower scores on right-hand and both-hand pegboard tasks than the healthy controls. Fast PSE-RAS also led to higher task scores when compared to no PSE-RAS, while no PSE-RAS had higher task scores than normal PSE-RAS. Faster upper-limb movements were then effectively induced with faster PSE-RAS.

The second study, also led by Wang et al. [53], investigated the use of PSE-RAS for functional movement training in individuals with psychotic-like experiences (PLEs) who may be at future risk of developing a psychotic disorder. The study included individuals with PLEs (*n* = 17) who were age and gender matched to healthy controls (*n* = 18), identified using the Prodromal Questionnaire and Community Assessment of Psychic Experiences, distributed in secondary schools and universities. Over 21 days, the participants completed 40 min daily functional upper-extremity task training of grasping and placing beans into bowls placed at varying positions. Participants with PLEs were then randomly assigned to either the PSE-RAS experimental group (*n* = 8) or the no-PSE-RAS control group (*n* = 9). After assessing the baseline movement speed, PSE-RAS was applied at normal, quick, and fast tempi while completing the task moving one bean per metronome beat. The control group performed the same task without PSE-RAS. Functional movement was analyzed using motion capture kinematic parameters, as well as normalized movement time and normalized number of movement units to assess the extent of movement slowing and irregular muscle contraction. Those with PLEs who received PSE-RAS had a reduced normalized movement time, number of movement units, and irregular muscle contraction at post-test than those who received no PSE-RAS. Both studies by Wang et al. [39,41] demonstrated the benefit of PSE for movement speed among psychiatric populations.

## 4. Discussion

This review synthesized 15 empirical papers assessing PSE for motor function across clinical populations, demonstrating that there is limited empirical research in this area. Even more limited is the research with PSE as the sole intervention. Of the included studies, three studies [40,41,42] used “PSE” as an independent intervention or condition, and two studies [43,46] used combined NMT motor applications. The remaining studies used PSE principles mislabeled, as one study [49] used “melodic auditory cueing,” one study [44] used “sonified cueing,” and eight studies [45,47,48,50,51,52,53,54] used PSE-RAS for UEs. In addition, the interventions varied significantly across the studies in respect to the musical stimuli used, personnel involved, dose, etc.

### 4.1. Efficacy of PSE on Motor Function

The use of PSE was found to be efficacious for motor function in children with Spastic Diplegia related to CP, as both Peng et al. [41] and Wang et al. [40] demonstrated motor improvements, particularly in movement smoothness, speed, and power, and gross motor capacity. Both studies used the same principles of individually composed PSE music that appeared to focus primarily on the movement timing and number of beats. This personalized approach to the exercise may have been an important factor contributing to its efficacy.

Among stroke populations, PSE has demonstrated its efficacy in improving motor outcomes across the included studies. Notably, these studies referred to the intervention using various terms, including “patterns of enhancement,” “musical cueing,” “rhythmic auditory-motor entrainment,” and “rhythmic auditory stimulation” of the upper extremities. Kang et al. [49] utilized three study conditions, but did not clarify whether the order of conditions was randomized to minimize the potential order effects. Factors like participant fatigue, learning, or expectations could have introduced bias, potentially compromising the study’s internal validity. As a result, the findings may not accurately reflect the true impact of auditory cueing on motor function. Malcolm et al. [51] showed positive UE effects; however, the small sample size (*n* = 5) and absence of a control group limit the generalizability of the findings. Kalidasan et al. [52] found PSE-RAS and mirror therapy to be beneficial when compared to CT, but PSE-RAS was demonstrated as the most effective intervention for hand function and grip, with Tian et al. [48] demonstrating the efficacy of both PSE-RAS and CT. Improvements in functional movement, particularly of the elbow were demonstrated in three of these papers, with a benefit of PSE-RAS during arm reaching [50,51] as well as an improvement in elbow flexion [48]. This may be supported by the findings of Chouhan et al. [47] who found a faster improvement among gross motor skills over fine. Future investigation may be helpful to determine if specific cue types are more beneficial for the movement type.

In PD, PSE has had mixed results on the motor outcomes in the three included studies but may benefit movement smoothness. Smith et al. [44] found that sonified PSE may have a positive effect on the movement smoothness over rhythmic PSE, while Bukowska et al. [46] found that NMT motor techniques may result in improvements of rhythmic movements, but not necessarily static stability. Fan et al. [45] also found a positive effect of PSE-RAS, as it resulted in increased movement speed. This inconsistency may demonstrate that the movement type, rhythmic or volitional, may be an important consideration when designing interventions. The discrepancies in the results may be due to the differences in sample size between the studies. Additionally, the study by Bukowska et al. [46] utilized a combination of NMT motor techniques, including PSE, TIMP, and RAS. While they found that NMT motor techniques may improve rhythmic movements, which is consistent with the existing knowledge on the influence of the rhythmic input on the motor output, the inclusion of multiple interventions makes it difficult to draw specific conclusions about the efficacy of PSE alone for the purposes of this review. It also appears that there was no randomization used in this study. While Fan et al. found an effect on movement speed, it may be of interest to further investigate the movement quality. Future rigorously designed, well-powered studies are warranted to draw conclusions on the efficacy of PSE in this population.

Using PSE for older adults had inconsistent efficacy results in the literature. Toma et al. [43] found an increase in muscle strength across all participants, with the majority showing improvements from pre- to post-. However, these results should be interpreted with caution due to the small sample size of only six participants, the absence of statistics, and an unspecified music therapy protocol, as PSE was integrated into a broader 60 min session with the opening and closing 15 min being PSE, and the middle 30 min being undisclosed. Although the PSE tasks included hand grip and arm movements (up and down), it remains unclear whether the central portion of the session focused on additional motor tasks or addressed different aspects of health. This lack of clarity makes it difficult to fully understand the structure of the therapy protocol and hinders the ability to definitively attribute improvements solely to PSE.

The literature suggests that in addition to facilitating motor improvements, PSE can also be used to support the motor maintenance of older adults. However, O’Konski et al. [42] found no significant effects of PSE on exercise outcomes. This raises the question of whether PSE contributes to long-term motor function maintenance. The duration and intensity of exercise exposure may help explain these findings, as this study conducted only eight sessions of 20 min each.

Another key consideration for this study is the impact of an imposed, clinician-selected tempo. While implementing individualized PSE clinically, cue synchronization can be adapted to different movement speeds by responding to different beats in the pulse structure, appropriate for their specific abilities. However, in a group setting, this becomes much more challenging. It is unclear if the facilitating clinician chose tempi based on the average group ability, the participant with the greatest needs, or the most able participant, making it difficult to ensure that the needs of all participants are being met. An imposed tempo then may not demonstrate the full breadth of PSE benefits possible for each group member. This may be a key consideration for both movement speed and, more importantly, movement quality for the study by O’Konski et al. [42] finding a lack of intervention efficacy.

PSE for psychiatric populations, a new area of investigation, was found to be effective, particularly for movement speed in both studies by Wang et al. [53,54].

Therefore, PSE for motor function demonstrated benefits for CP, stroke, PD, and psychiatric conditions, though inconsistencies were noted in geriatric populations.

### 4.2. The Efficacy Across Clinical Populations

When PSE’s efficacy for motor function was analyzed across clinical populations, this review of the literature noted some key considerations. While the studies with positive outcomes employed various measures and focused on different movement types, they consistently support PSE as an effective approach for improving motor function. However, to date, only a small number of studies in each population have been conducted, which presents noticeable limitations.

PSE research has utilized various study designs. Four RCT studies using PSE were conducted with CP [40], stroke [47,55], and psychiatric [53] populations. The pattern of findings demonstrate a positive effect on gross motor skills, assessment scores, muscle contraction and co-activation, and movement speed. While these results across populations are promising, there were inconsistencies in the design and clinical populations. Of these studies, the PSE interventions were purely rhythmic and metronomic when implemented among stroke and psychiatric populations, while the study with Cerebral Palsy used pre-recorded PSE pitched music. Three of the studies [40,48,53] had study timelines spanning 3–6 weeks, while one [47] held a single-session study. Between-subjects studies demonstrated improved movement smoothness, speed, and stability [44,45,46,53], and were all conducted with PD populations. Within-subject studies showed improvements in movement quality, coordination, smoothness, and speed [41,49,50,53], with one study showing no significant improvements [42]. Pre–post studies had a wide variety of positive outcomes, demonstrating improvements in muscle strength [43], hand function and grip [43,52], trunk compensation [51], functional movements of the shoulder, elbow, and hand [51,52], and movement speed [51]. No studies implemented a follow-up or retention test, making it difficult to determine if the effects of PSE are long-lasting.

Two of the included studies shared an overlapping study design in differing populations, with Smith et al. [44] focusing on PD and Kang et al. [49] studying stroke. Both studies used three cueing conditions: no cue, rhythmic PSE, and melodic/sonified PSE. However, terminology inconsistency was present, as Smith et al. referred to it as “sonified cueing,” while Kang et al. used the term “musical auditory cue.” Despite both using similar kinematic outcome measures and overlapping cueing conditions, they yielded different results. In the PD group, melodic PSE improved movement smoothness, while in the stroke group, rhythmic PSE led to enhanced movements across all the parameters.

Similarly, three other studies utilized similar PSE-RAS experimental designs across populations. The studies by Fan et al. [45] and Wang et al. [53,54] implemented PSE-RAS at a baseline speed with increasing tempo, typically moving from 100% to 110% to 120%. Fan et al. utilized this design among PD while both Wang et al. studies focused on psychiatric populations. Fan et al. [45] and Wang et al. [54] both demonstrated a positive condition effect on right hand scores. Each study concluded that faster PSE-RAS led to improvements in the task speed and scores and movement slowing. Additional studies utilized a rhythmic PSE or PSE-RAS intervention with the metronome [47,48,49,50,51,52]. While these studies addressed stroke populations with positive results, they differed in their designs, outcome measures, and target movements. These studies showed that using a metronome during rhythmic PSE or PSE-RAS resulted in faster improvements in gross motor skills over fine [47], improvements in biceps and/or triceps control [48,50], improved movement function, quality, or coordination [49,50,51,52], improved elbow extension [50,51], decreased movement time [50,51], decreased number of movement units [50], decreased trunk compensation [51], improved shoulder flexion [51], and improved hand grip [52]. This may then contribute to the importance of rhythmic interventions and PSE in stroke recovery. Of the included studies, six used pre-recorded pitched musical stimuli [40,41,42,44,46,49] while only one used live musical stimuli [43]. The studies that used pre-recorded musical stimuli demonstrated some positive effects of PSE, including improved gross motor capacity [40], movement smoothness [41,44], improved movement time [41,49], and improved stability [46], except for the study by O’Konski et al. [42] which found that PSE did not have any significant effect. The study using live music demonstrated a positive impact of PSE on muscle strength [43], though this used minimal outcome measures for motor function. However, it remains difficult to compare any effect of live versus pre-recorded music across these studies, as the outcome measures, target movements, dose, and populations were varied.

### 4.3. Potential Neural Mechanisms Underlying PSE

Dynamical systems theory offers a potential framework for the neural mechanisms underlying PSE. Musical rhythm can entrain oscillatory activities, particularly in motor-associated areas of the brain. Upper-limb movements consist of regular submovements that occur at oscillatory frequencies between 1 and 4 Hz, which are found in the motor and motor-associated cortical areas [55]. These oscillations may encode both high-level parameters (e.g., reach direction) and low-level parameters (e.g., force or muscle activity) [56].

Neural phase locking to the musical pulse appears preserved in older adults, suggesting the potential for rhythm-based interventions to support health functions [57]. Clinically, RAS has been show to enhance motor function, particularly in PD by modulating brain oscillations [58]. These mechanisms may also be active during PSE, underscoring the potential for functional change. Further investigation into other musical elements—such as pitch, melody, timbre, and dynamics—may offer deeper insight into the underlying neural mechanisms and enrich our understanding of how much they influence brain function.

### 4.4. Limitations

The key limitation of this literature search was the inconsistency in the terminology surrounding PSE. We encountered several different terms for interventions that are PSE in principle, including “PSE,” “RAS for upper extremities,” “rhythmic auditory cueing,” “sonified cueing,” “melodic auditory cueing,” and “patterned sensory enhanced music.” This variation in classification makes it challenging to accurately synthesize the information and may result in relevant studies being overlooked or missed; however, we aimed to include those that incorporated both rhythmic or musical interventions for motor function. This scoping review was of the highly heterogeneous literature, including differences in musical stimuli, intervention dosage, study design, and sample size. It may then be beneficial for future systematic reviews to provide quality assessment and critical appraisal of the literature across different domains and designs. Additionally, the number of studies included in this review were small, making it difficult to draw substantial conclusions per population. Continued development in this area of research will contribute to the growing strength of the generalizations across populations.

### 4.5. Future Directions

This review has identified some considerations that can be incorporated into future study designs to fully understand the efficacy of PSE in relation to motor function. Methodological limitations of reviewed studies include small sample sizes, insufficient details on the interventions, use of combined therapies that obscure the unique effects of PSE, lack of statistical analyses, investigation of only immediate effects, and absence of longer-term outcome assessments. Thus, more rigorous study designs are warranted. Advancement of the research and practice of PSE would benefit from increased well-powered randomized controlled trials, neuroimaging and neurophysiological studies on the underlying neurobiological mechanisms, comparative studies to identify the effects of different musical elements on movement, analyses of different types of music to assess motivation, use of consistent terminology to define PSE, and clinical training in Neurologic Music Therapy.

More specifically, the importance of rhythm in motor training has been well-demonstrated. However, one of the aspects of PSE that differentiates it from RAS and other rhythmic cueing interventions is the incorporation of additional musical elements, like melody, harmony, and form. While Kang et al. [49] suggested that the effect of pitch in addition to rhythm may have benefited shoulder function and movement time, there lies potential for further investigation. It may be of interest for future comparative studies to explore whether the musical elements of PSE, beyond rhythm alone, contribute to motor outcomes, or if it is primarily the rhythmic component of PSE that drives the observed changes. Additionally, some studies presented in this review provided limited detail on the actual musical intervention or stimuli that were used. It may be beneficial for future studies to include this level of detail to ensure its reproducibility and replicability and that it follows the principles of PSE.

Furthermore, the use of auditory cues for specific movement types may benefit from further investigation as intrinsically rhythmic movements may require different cues compared to volitional ones. Much of the research on rhythmic cueing has focused on inherently rhythmic movements like gait, suggesting that cue effectiveness may depend on the movement type [59,60,61]. For example, sensorimotor synchronization appears to influence discrete movements more than rhythmic ones. For example, Smith et al. [44] found that rhythmic cueing did not improve upper-extremity movement smoothness in individuals with Parkinson’s Disease, while Bukowska et al. [46] observed gait improvements but no changes in static stability after an NMT motor protocol. These findings may stem from movement differences, indicating that NMT techniques may impact rhythmic more than non-rhythmic movements. It may be beneficial for future investigations to determine how musical, rhythmic, and auditory cueing transfer to volitional upper-extremity movements.

Further investigation into the appropriate PSE dosage and treatment frequency may also be beneficial to ensure that it optimizes the motor outcomes, supports rehabilitative goals, and aligns with the existing physical activity guidelines for various clinical populations. Additionally, to determine any lasting effects of PSE, future studies may benefit from the inclusion of retention tests or follow-up assessment measures.

Motivation plays a critical role in exercise repetition, and music can serve as a powerful motivator through mood enhancement [62] which may potentially increase the exercise frequency. Motor rehabilitation is most effective when it involves a high repetition, intensity, dose, frequency, challenge, and duration [63,64]. Given the potential monotony of some rehabilitation exercises, patient motivation becomes essential for skill reacquisition, progression, and maintenance. Highly motivated patients often view rehabilitation as the key to their recovery [65], highlighting the need for interventions that maximize motivation.

Motor maintenance goals may also be a consideration factor. Particularly among older adults, those with higher physiological fitness tend to have a lower mortality risk [66,67], and maintaining a physically active lifestyle throughout middle and older adulthood is linked to better health and longevity [68,69,70]. To optimize motor outcomes and maintenance, older adults are advised to exercise near their maximum capacity and aim for at least 150 min of physical activity per week [71,72]. Future research may benefit from using both cross-sectional and longitudinal study designs to assess PSE’s impact on health and longevity, as well as the optimal PSE dose for geriatric populations. It would be valuable to further examine the duration of PSE exposure in these studies and investigate the optimal dose of PSE to ensure that it aligns with the existing physical activity guidelines for various clinical populations.

Personalized music compositions and individual music preferences have been emphasized as crucial factors for effective PSE, and promote feelings of well-being [43]. Music can increase motivation when compared to no music, leading to improved clinical outcomes [60]. For example, PSE led to anecdotal reports of enjoyment of and a preference for music sessions, which may boost motivation and exercise repetition [73]. Preferred music may also potentially foster greater engagement [74,75], and live versus pre-recorded music may have an impact on outcomes [76,77]. It is important for future studies to administer a questionnaire on motivation to correlate with interventional outcome measures.

## 5. Conclusions

There is growth in the application of music interventions for improving health and rehabilitation outcomes, including PSE. This review aimed to synthesize the existing literature on PSE as a therapeutic intervention across clinical populations, and suggests that it is effective for improving motor function for CP, stroke, PD, and psychiatric conditions, with inconsistencies demonstrated in geriatrics. Future research may benefit from a focus on specific musical elements, movement type, dosage, the role of motivation, and music preference. Using consistent terminology to differentiate between “PSE” and other musically cued interventions (e.g., “RAS”) should be considered important in the move towards the standardization of each technique.

While much of the current research has concentrated on music interventions for stroke and PD, the exploration of such interventions for other populations, including those with brain injuries, multiple sclerosis, Huntington’s Disease, Developmental Coordination Disorder, and Autism Spectrum Disorder, remains limited. The growing demands placed on healthcare systems underscores the need to optimize the clinical techniques to effectively address patient needs. Thus, further research is essential to fully understand the potential benefits of music-based interventions, including motor-focused interventions like PSE.

## Figures and Tables

**Table 1:** Summary of included studies.

Author(s), Year	Clinical Population	Study Design	Sample Size (*N*) Age (M ± SD) Sex (F/M)	PSE Intervention Design				Key Findings
				Movement Task(s)	Comparison Task(s)	Musical Stimuli	Duration	
Wang et al., 2013 [40]	Cerebral Palsy–Spastic Diplegia	RCT	36 PSE: 18, 9 ± 1.99 (6 F/12 M) Controls: 18, 8.98 ± 2.61 (3 F/15 M)	Loaded sit-to-stands (LSTS)	-PSE: PSE music during LSTS -Controls: No music during LSTS	-Pre-recorded keyboard music -Individual, familiar, and preferred music -Spatial cues: ascending and descending melodic lines and volume -Temporal cues: changing meter and tempi for different movement aspects -Force cues: rhythm and articulation during seat-off transfer	Three sets of 10 repetitions, 3 times/week for 6 weeks	PSE ↑ gross motor capacity, but not significantly in daily functioning, strength, and walking speed
Peng et al., 2011 [41]	Cerebral Palsy–Spastic Diplegia	Pre-, post-test	23 8.7 ± 2 (10 F/13 M)	LSTS	-PSE Condition: PSE music during LSTS -Control Condition: No music during LSTS	-Pre-recorded keyboard music -Individual music -Spatial cues: ascending and descending melodic lines and volume -Temporal cues: tempo based on baseline speed; simple harmony for synchronization -Force cues: rhythm and articulation during seat-off transfer; increased volume during sitting; decreased volume during standing	Single session: two trials of eight repetitions	PSE ↑ LSTS immediately, ↑ total/knee peak extensor powers, ↑ movement smoothness, ↓ speed
O’Konski et al., 2010 [42]	Geriatrics	Pre-, post-test	45 73.5 ± 20.5 (42 F/3 M)	Seated exercise	-PSE Condition: PSE music during exercise -Background Music Condition: big band music during exercise	-Pre-Recorded PSE music -Pre-recorded big band music	Three 20 min sessions of each condition	No significant differences between conditions
Toma et al., 2024 [43]	Geriatrics–Neuromotor Deficits	Pre-, post-test pilot	6 73.8 (2 F/4 M)	Hand grip, arms up and down	-PSE music during exercise	-Live PSE music -Simultaneous with metronome -Spatial cues: ascending and descending melodic lines during arms up and down -Temporal cues: exercises synchronized to a set tempo -Force cues: tense harmony during hand grip, resolution during relaxation	Two 30 min sessions/week for 4 weeks	PSE ↑ muscle strength
Smith et al., 2024 [44]	Parkinson’s Disease	CCT	17 PD: 7, 74.9 ± 4.43 (15 F/8 M) Controls: 10, 19.9 ± 0.74 (7 F/3 M)	Repetitive arm reaching	-Three cueing conditions: No cueing, Rhythmic PSE, Sonified PSE	-Rhythmic PSE: metronome only at 70 beats per minute -Sonified PSE: pre-recorded, familiar piano folk tune simultaneous with metronome at 70 beats per minute -Spatial cues: forward movement cued with a chord on strong beats with loud volume; backward movement on weak beats with soft volume -Temporal cues: reaching synchronized to beat -Melody played with strong chordal cues to emphasize extension and flexion	Three 60 s trials of each condition	Sonified PSE ↑ movement smoothness
Fan et al., 2022 [45]	Parkinson’s Disease	CCT	46 PD: 23, 67.30 ± 7.86 (15 F/8 M) Controls: 23, 64.13 ± 5.59 (13 F/10 M)	Pegboard task	-PSE-RAS during a pegboard task at 100% speed (self-paced baseline), 110%, and 120% with left hand, right hand, and both	-PSE-RAS -Metronome only -Temporal cues: movement synchronized to beat at each speed	30 s per task	Faster PSE-RAS ↑ movement speed for each task
Bukowska et al., 2016 [46]	Parkinson’s Disease	CCT pilot	55 NMT: 30, 63.4 ± 10.61 (15 F/15 M) Controls: 25, 63.44 ± 9.67 (10 F/15 M)	Activities of daily living, balance, pre-gait, and gait	-NMT: PSE, RAS, and TIMP for activities of daily living, balance, pre-gait, and gait -Controls: maintain current ADLs with no music	-Pre-recorded rhythmic music -Mostly African and Indian music -With embedded metronome -Temporal cues: rhythm and beat synchronized movement -General use of musical elements—pitch, dynamics, harmony, meter, tempo, and rhythm to organize movement	Four 45 min sessions/week for 4 weeks	NMT ↑ rhythmic movements and ↑ in stability with eyes closed/proprioception
Chouhan et al., 2012 [47]	Stroke	RCT	45 PSE-RAS: 15, 56.73 ± 5.99 (3 F/12 M) VC: 15, 58.13 ± 4.14 (3 F/12 M) CT: 15, 57.33 ± 5.51 (3 F/12 M)	Affected arm/hand reaching/functional tasks	-PSE-RAS: affected arm/hand reaching/functional tasks self-paced then with PSE-RAS + CT -VC: select, lift, and transfer different objects + CT -CT: stretching of tightened muscles and exercises	-PSE-RAS -Metronome only -Temporal cues: movement synchronized to beat	30 s trials for each task, 5–10 repetitions	All groups had ↑ in FM-UE. Gross motor skills ↑ faster than fine motor
Tian et al., 2020 [48]	Stroke	RCT pilot	30 PSE-RAS: 15, 66.67 ± 13.59 (2 F/13 M) Controls: 15, 64.40 ± 13.41 (5 F/10 M)	Shoulder/arm/hand movements and functional tasks	PSE-RAS during shoulder/arm/hand movements and functional tasks at the baseline speed, increasing by 5%	-PSE-RAS -Metronome only -Temporal cues: movement synchronized to beat	PT/OT 30 min/day each, 5 days/week for 4 weeks PSE-RAS: additional PSE-RAS 30 min/day Controls: additional PT/OT 15 min/day each	PSE-RAS ↑ WMFT and ↑ BI. ↑ co-activation interval of biceps and triceps
Kang et al., 2020 [49]	Stroke	Pre-, post-test	18 49.78 ± 15.55 (8 F/10 M)	Affected shoulder movements	-During rhythmic PSE and melodic PSE (the same time pattern, different melodic frequencies)	-Rhythmic PSE: metronome only -Spatial cues during melodic PSE: ascending, stationary, and descending melodic lines for shoulder abduction, holding, and adduction -Temporal cues: movement synchronized to beat in both cueing conditions	Single session: five trials per cueing condition	Rhythmic and melodic PSE ↑ all movements. Melodic PSE ↓ movement time
Kim et al., 2014 [50]	Stroke	Pre-, post-test	16 49.2 ± 17.65 (7 F/9 M)	Affected arm reaching	-First at a self-paced speed, then with PSE-RAS at a matched baseline speed	-PSE-RAS -Metronome only -Temporal cues: movement synchronized to beat	Single session: 1 min task intervals	PSE-RAS ↑ movement quality and coordination, ↑ elbow extension, ↓ movement time and number of units, ↑ tricep activation, ↓ co-contraction ratio
Malcolm et al., 2009 [51]	Stroke	Pre-, post-test pilot	5 72.8 ± 6.5 (0 F/5 M)	Affected arm reaching with changing distances and directions	-First at a self-paced speed, then with PSE-RAS at a matched baseline speed	-PSE-RAS -Metronome only -Temporal cues: movement synchronized to beat	2 weeks of 3 days/week: 1 onsite + 2 home hours + 2 days/week: 3 home hours	PSE-RAS ↓ trunk compensation, ↑ shoulder flexion, ↑ elbow extension, ↑ movement time, ↑ velocity, ↑ functional gains
Kalidasan et al., 2022 [52]	Stroke	Pre-, post-test	60 35–60 years PSE-RAS: 20 MT: 20 CT: 20	Affected arm/hand functional tasks	-PSE-RAS: affected arm/hand functional tasks with PSE-RAS -MT: simultaneous affected and unaffected arm/hand movements with mirror -CT: free arm movements, tone normalization, voluntary sensory re-education	-PSE-RAS -Metronome only -Temporal cues: movement synchronized to beat	20 min/day, five sessions/week for 4 weeks	PSE-RAS had most ↑ hand function and ↑ hand grip
Wang et al., 2023 [53]	Psychiatric Conditions–Psychotic-Like Experiences	RCT	35 PLEs: 17, 20.71 ± 2.45 (9 F/8 M) Controls: 18, 21.22 ± 4.71 (8 F/10 M) → PLEs with PSE-RAS: 8 PLEs without PSE-RAS: 9	Reach/grasp/move beans	-PLEs with PSE-RAS: functional tasks of reach/grasp/move beans at normal, quick, and fast tempi -PLEs without PSE-RAS: Functional task of reach/grasp/move beans as fast as possible	-PSE-RAS -Metronome only -Temporal cues: movement synchronized to beat	40 min/day for 21 days	PSE-RAS ↓ movement slowing, ↓ number of movement units, ↓ irregular muscle contraction
Wang et al., 2021 [54]	Psychiatric Conditions–Schizophrenia Spectrum Disorders	CCT	60 SSD: 30, 47.77 ± 11.54 (13 F/17 M) Controls: 30, 40.43 ± 14.74 (15 F/15 M)	Pegboard task	-Normal PSE-RAS Condition: pegboard task at 100% of the baseline speed -Fast PSE-RAS Condition: pegboard task at 120% of the baseline speed	-PSE-RAS -Metronome only -Temporal cues: movement synchronized to beat	Single session: three trials of each condition	Fast PSE-RAS ↑ movement speed and ↑ task scores

Note: ADLs: activities of daily living, BI: Barthel Index, CCT: clinical control trial, CT: conventional therapy, FM-UE: Fugl-Meyer Upper Extremity, F: female, LSTS: loaded sit-to-stands, M: male, MT: mirror therapy, NMT: neurologic music therapy, OT: occupational therapy, PD: Parkinson’s Disease, PLEs: psychotic-like experiences, PT: physiotherapy, PSE: patterned sensory enhancement, RAS: rhythmic auditory stimulation, RCT: randomized controlled trial, SSD: schizophrenia spectrum disorders, TIMP: therapeutic instrumental music playing, VC: visual cueing, WMFT: Wolf Motor Function Test.

## Data Availability

Not applicable.

## Supplementary Materials

The following supporting information can be downloaded at: https://www.mdpi.com/article/10.3390/brainsci15070664/s1, Table S1: Search Strategy. Table S2: Exclusion Table. References [78,79,80,81,82,83,84,85,86,87,88] are cited in Table S2.
