# Supplementary material for: The Effects of Music-Based Patterned Sensory Enhancement on Motor Function: A Scoping Review
Source: Brain Sci. 2025 Jun 20;15(7):664. doi: 10.3390/brainsci15070664 (PMC12293833; doi:10.3390/brainsci15070664)
Supplement: Supplementary file 1 [file brainsci-15-00664-s001.zip › brainsci-3643077-Caputo Supplementary Table S1.pdf]

Supplementary Table S1. Search Strategy.

**Ovid MEDLINE**	
1	Patterned Sensory Enhancement.mp. 6
2	Pattern* Sensory Enhance*.mp. 8
3	music based exercise.mp. 2
4	rhythmic cue*.mp. 112
5	(auditory adj3 rhythmic cue*).mp. [mp=title, book title, abstract, original title, name of substance word, subject heading word, floating sub-heading word, keyword heading word, organism supplementary concept word, protocol supplementary concept word, rare disease supplementary concept word, unique identifier, synonyms, population supplementary concept word, anatomy supplementary concept word] 14
6	(rhythmic auditory adj3 cue*).mp. [mp=title, book title, abstract, original title, name of substance word, subject heading word, floating sub-heading word, keyword heading word, organism supplementary concept word, protocol supplementary concept word, rare disease supplementary concept word, unique identifier, synonyms, population supplementary concept word, anatomy supplementary concept word] 110
7	live music supported exercise.mp. 1
8	music supported exercise.mp. 1
9	1 or 2 or 3 or 4 or 5 or 6 or 7 or 8 219
10	limit 9 to humans 162

**Embase Classic+Embase**	
1	Patterned Sensory Enhancement.mp. 17
2	Pattern* Sensory Enhance*.mp. 19
3	music based exercise.mp. 2
4	rhythmic cue*.mp. 141
5	(auditory adj3 rhythmic cue*).mp. [mp=title, abstract, heading word, drug trade name, original title, device manufacturer, drug manufacturer, device trade name, keyword heading word, floating subheading word, candidate term word] 21
6	(rhythmic auditory adj3 cue*).mp. [mp=title, abstract, heading word, drug trade name, original title, device manufacturer, drug manufacturer, device trade name, keyword heading word, floating subheading word, candidate term word] 152
7	live music supported exercise.mp. 1
8	music supported exercise.mp. 2
9	1 or 2 or 3 or 4 or 5 or 6 or 7 or 8 290
10	limit 9 to human 274

**APA PsycInfo**
1 Patterned Sensory Enhancement.mp. 8<br>2 Pattern* Sensory Enhance*.mp. 10<br>3 music based exercise.mp. 1<br>4 rhythmic cue*.mp. 71<br>5 (auditory adj3 rhythmic cue*).mp. [mp=title, abstract, heading word, table of contents, key concepts, original title, tests & measures, mesh word] 7<br>6 (rhythmic auditory adj3 cue*).mp. [mp=title, abstract, heading word, table of contents, key concepts, original title, tests & measures, mesh word] 44<br>7 (live adj3 music supported exercise).mp. [mp=title, abstract, heading word, table of contents, key concepts, original title, tests & measures, mesh word]1<br>8 1 or 2 or 3 or 4 or 5 or 6 or 7 117<br>9 limit 8 to human 115

**SCOPUS**
TITLE-ABS-KEY ({patterned sensory enhancement} OR {music based exercise} OR "Pattern* Sensory Enhance*" OR "rhythmic cue*" OR "rhythmic auditory cue*" OR {music supported exercise})<br>277 results

**CINAHL**
“music supported exercise” OR “rhythmic auditory stimul*” OR “rhythmic auditory cue*” OR “rhythmic cue*” OR “music based exercise” OR “Pattern* Sensory Enhance*”<br>163 results
