# Supplementary material for: The Effects of Music-Based Patterned Sensory Enhancement on Motor Function: A Scoping Review
Source: Brain Sci. 2025 Jun 20;15(7):664. doi: 10.3390/brainsci15070664 (PMC12293833; doi:10.3390/brainsci15070664)
Supplement: Supplementary file 1 [file brainsci-15-00664-s001.zip › brainsci-3643077-Table S2 rev.pdf]

Table S2: List of studies initially considered, later excluded (also see lists of *Eligibility criteria* in main paper).

**Author(s), Year**	**Title**	a	b	c
Kang et al., 2023 [79]	Neurologic music therapy combined with EEG-tDCS for upper motor extremity performance in patients with corticobasal syndrome: Study protocol for a novel approach		✓	
McLean et al., 2022 [80]	Combining music therapy techniques with physical therapy in an individual following brain injury: A case study in the use of both patterned sensory enhancement and rhythmic auditory stimulation		✓	
Snyder et al., 2022 [81]	Patterned sensory enhancement for improving sit-to-stand transfer performance in children & adolescents with acquired brain injury: A case series		✓	
Kim, et al., 2019 [82]	Patterned sensory enhancement (PSE) music for upper limb function changes in children with spastic cerebral palsy	✓		
Jia, et al., 2017 [83]	Spasmodic hemiplegia after stroke treated with scalp acupuncture, music therapy and rehabilitation: a randomized controlled trial	✓		
Wang et al., 2012 [84]	A randomized controlled trial of neurologic music therapy during a functional strengthening program for children with cerebral palsy		✓	
Thompson & Weintraub, 2013 [85]	The clinical use of neurologic music therapy in a brain injury rehabilitation setting: A retrospective analysis of therapeutic practice		✓	
Clark et al., 2012 [86]	Patterned sensory enhancement: An innovative neurologic music therapy intervention for older adults during rehabilitation		✓	
Wang et al., 2011 [87]	Long-term effects of therapeutic music combined with loaded sit-to-stand resistance exercise on mobility for children with cerebral palsy		✓	
Whitall & Mccombe Waller, 2013 [88]	Does the use of an auditory cue facilitate the motor control and contribute to the rehabilitation of upper extremity movements after stroke?		✓	
Barta et al., 2019 [89]	Validity and inter-rater reliability of the SOAR tool during ambulation in individuals with Parkinson disease			✓

Note: **a:** no English translation; **b:** wrong study design; **c:** did not report efficacy of PSE
